# Progress of Our Principles

**Published:** 1855-11

**Authors:** 


### PROGRESS OF OUR PRINCIPLES.

In the first number of the *Journal*, we announced one of our objects to be, *The Prevention of Disease without the use of Medicinal Means;* that such was the highest aim of the good and great physician, and our readers will bear us witness, that we have never offered them a single medicinal recipe, except in case of a severe attack of cholera, when no physician could be had; that our uniform course has been to instruct them as to the best method of *maintaining* health through the instrumentality of the daily habits of life, as to eating, sleeping, dress, exercise, &c. Since then our mottoes have found utterance from the people, while the press with her thousand tongues has given its hearty and intelligent approbation. We feel quite sure, that no medical journal ever published in this country has met with a warmer welcome wherever it has found its way, while our patrons, in renewing their subscriptions, are almost extravagant in their commendations. When our first number was issued without a subscriber, we did not even aim to the high approbation which the *Journal* has already secured. Our present wish and aim is, that no word, uttered through our pages, shall forfeit that valued approbation.

May we ask here, that each subscriber feel sufficient interest in extending to their most loved friends the pleasure and profit which they themselves have so often derived from the pages of the *Journal*, as to send other names besides their own, when they renew their subscriptions for the coming year, thus making themselves co-laborers with us in a work which is at once philosophical and humane, and whose progress is evidenced by the fact that the Treasurer of the Massachusetts Medical Society announced, at the last meeting, that he had received the sum of *one hundred dollars* from a member of the Society, for a prize for 1857, on conditions similar to those of 1856—on the following theme:—" We would regard every approach towards the rational and successful prevention and management of disease, without the necessity of drugs, to be an advance in favor of humanity and scientific medicine."

